# Intraoperative global longitudinal strain and strain rate as predictors of unfavorable outcome following on-pump mitral surgery: a prospective observational study

**DOI:** 10.1186/s44158-025-00288-1

**Published:** 2025-10-22

**Authors:** Fabrizio Monaco, Alessandra Bonaccorso, Jacopo D’Andria Ursoleo, Alessandro Pruna, Caterina Cecilia Lerose, Ambra Licia Di Prima, Gaia Barucco, Giovanni Landoni, Margherita Licheri, Mit-Go Study Group Collaborators

**Affiliations:** 1 Department of Anesthesia and Intensive Care, IRCCS San Raffaele Scientific Institute, Via Olgettina 60, Milan, 20132 Italy; 2 Department of Paediatrics and Neonatal Intensive Care Unit, AOU S. Anna, Ferrara, Italy; 3 School of Medicine, Vita-Salute San Raffaele University, Milan, Italy

**Keywords:** Cardiopulmonary bypass, Global longitudinal strain, Low cardiac output syndrome, Patient outcomes, Mitral surgery, Strain rate, Transesophageal echocardiography

## Abstract

**Background:**

Low cardiac output syndrome (LCOS) is a life-threatening complication following cardiac surgery. We explored the predictive value of transesophageal echocardiography (TEE)-measured global longitudinal strain (GLS) and strain rate (SR) to establish clinically relevant thresholds for both GLS and SR to subsequently develop a predictive model for LCOS.

**Methods:**

A trained anesthesiologist performed standard TEE after general anesthesia induction and before establishment of cardiopulmonary bypass. GLS and SR were obtained using 2D speckle tracking. The occurrence of LCOS after mitral surgery was the primary outcome. Predictive accuracy was assessed using receiver operating characteristic (ROC) curves and Youden index. A multivariable logistic regression model was internally validated via bootstrapping. Associations between GLS categories (< − 19.1 vs > − 19.1%) and outcomes were evaluated using inverse probability of treatment weighting (IPTW).

**Results:**

In 126 included patients, LCOS occurred in 31 (25%) instances. Optimal cut-offs to predict LCOS were > − 19.1% for GLS (area under the curve [AUC], 0.79; *P* < 0.001) and ≤ − 0.98 s^−1^ for SR (AUC, 0.66; *P* = 0.01). Predictors for LCOS were GLS > − 19.1%, tricuspid annular plane systolic excursion (TAPSE), ejection fraction (EF), and creatinine clearance (CrCl). The model showed strong performance (*R*^2^, 0.580; c-statistic, 0.898; optimism-corrected *R*^2^ = 0.517 and AUC 0.77). GLS > − 19.1% was also associated with time on mechanical ventilation (*P* = 0.015), length of ICU (*P* = 0.004), and hospital stay (*P* < 0.001). After IPTW-weighted analyses, patients with GLS > − 19.1% had significantly higher odds of developing postoperative LCOS (odds ratio, 5.48; 95% confidence interval, 1.63–18.5;* P* = 0.006).

**Conclusions:**

We found that GLS, TAPSE, EF, and CrCl were independent predictors of postoperative LCOS in patients undergoing mitral surgery. Among them, a GLS value > − 19.1% was associated with higher odds of LCOS.

**Clinical trial number:**

ClinicalTrials.gov, NCT04045340, date of registration: 02.08.2019.

**Supplementary Information:**

The online version contains supplementary material available at 10.1186/s44158-025-00288-1.

## Introduction

Low cardiac output syndrome (LCOS) is a major concern following cardiac surgery with the use of cardiopulmonary bypass (CPB) [1] and is characterized by a decline in cardiac output resulting in systemic hypoperfusion and inadequate oxygen delivery [2]. This complication is associated with increased morbidity and mortality, thus making its early detection and intervention crucial [3]. Several studies have identified risk factors for LCOS, which span patient advanced age, prolonged CPB time, urgent or emergent surgery, and impaired left ventricular function measured by left ventricular ejection fraction (LVEF) [4]. While LVEF still plays a pivotal role in risk stratification, it is not devoid of limitations (e.g., dependence on image quality and load/volume, operator subjectivity, limited reproducibility) [5]. In recent years, global longitudinal strain (GLS) evaluated using automated 2D speckle-tracking echocardiography (STE) has emerged as a promising alternative to overcome these limitations [6]. Unlike LVEF, which assesses changes in chamber volume, GLS and strain rate (SR) reflect the longitudinal deformation of the myocardium due to contraction and the rate of myocardial deformation over time, respectively, offering a more specific measure of myocardial function [7]. Moreover, GLS demonstrated better reproducibility and lower inter-observer variability compared to LVEF [8, 9].

While pre- and post-operative transthoracic echocardiography (TTE) using GLS has been extensively studied to predict postoperative recovery and inform optimal surgical timing, the role of perioperative transesophageal echocardiography (TEE) in predicting early and late outcomes remains underexplored. To date, no studies investigated the role of intraoperative GLS-TEE and SR-TEE as predictors of LCOS in mitral valve surgery. In this regard, mitral valve surgery patients are at heightened risk of developing postoperative cardiac dysfunction, mainly due to preoperative overestimation of their ejection fraction coupled with the occurrence of postoperative afterload mismatch [10]. This vulnerability underscores the need for improved methods to predict LCOS in this specific patient population.

Therefore, we designed a prospective observational study to investigate the potential of TEE-GLS and SR—acquired before CPB—in predicting LCOS in patients undergoing on-pump mitral valve surgery. The aims of this investigation were as follows: (i) identifying clinically adequate cut-off values for both GLS and SR; (ii) developing a multivariable prognostic model based on LCOS prediction for patients undergoing on-pump mitral valve surgery, which will serve as a tool to estimate individual patients’ risk of developing LCOS after surgery.

## Material and methods

### Study population and enrollment criteria

We conducted a prospective, single-center cohort study at IRCCS San Raffaele Scientific Institute in accordance with the Strengthening the Reporting of Observational Studies in Epidemiology (STROBE) statement (*Supplementary Material, Supplemental Table S1*) [11]. The study was approved by the IRCCS San Raffaele Scientific Institute Research Ethics Committee (Protocol number STS-CE 065/8) and written informed consent was obtained from all subjects participating in the study. This study was registered on the ClinicalTrials.gov website (NCT04045340) before enrollment started.

Inclusion criteria were as follows: patients older than 18 years, signed informed consent, undergoing elective on-pump mitral valve repair or replacement, high-quality pre-CPB TEE mid-esophageal views of the left ventricle (four chamber, two chamber, and long axis) at a minimum frame rate of 50 Hz. Exclusion criteria were as follows: urgent or emergent surgery, need for pre-procedural inotropes/vasopressors or mechanical circulatory support, preoperative presence of ventricular pacing as well as prior heart transplantation or implantation of a ventricular assist device, poor-quality echocardiographic images (defined as > 3 unacceptable myocardial segments in speckle-tracking echocardiographic analysis) and ECG artifacts, deep hypothermic arrest. Patients with severe multiple valvular defects underwent combined surgery. Of note, in patients with mild to moderate mitral regurgitation (i.e., grade 1 or 2), on-pump surgical correction was indicated if one or more of the following criteria were met: presence of symptoms (e.g., dyspnea, fatigue), evidence of structural abnormalities (e.g., atrial dilation), concomitant arrhythmias (e.g., atrial fibrillation), or signs of pulmonary hypertension on stress testing.

### Perioperative anesthetic and hemodynamic management

All patients scheduled to undergo on-pump mitral valve surgery received standard anesthesia monitoring protocols, encompassing invasive blood pressure, central venous pressure, and TEE monitoring. General anesthesia (GA) was induced with midazolam, propofol, fentanyl, and nondepolarizing muscle relaxants and maintained using fentanyl, sevoflurane or desflurane, and nondepolarizing muscle relaxants. Surgical procedures were performed through either a full midline sternotomy or a minimally invasive right mini-thoracotomy. CPB was established and intermittent antegrade crystalloid cardioplegia was administered during the surgery. Standardized protocols for heparinization and its reversal, as well as CPB initiation and separation, were performed in accordance with institutional protocols [12]. After surgery, all patients were admitted to the cardiac ICU and followed the usual care institutional protocols until their discharge from hospital.

### Echocardiography

Intraoperative TEE was performed using a Philips X7/X8 probe connected to an EPIQ 7 ultrasound system (Philips Medical, Brussels, Belgium) and 2D speckle tracking echocardiography (2D-STE) to assess left ventricular function by experienced cardiothoracic anesthesiologists certified by the National Board of Echocardiography in Perioperative Transesophageal Echocardiography, adhering to the guidelines established by the American Society of Echocardiography and the European Association of Cardiovascular Imaging [13]. Images were obtained after the induction of GA and prior to heart cannulation, in patients who were mechanically ventilated and hemodynamically stable. The cardiac anesthesiologists who evaluated the echocardiographic images were blinded to the outcomes and were not involved in the management of patients in the ICU. GLS and SR at peak systole were computed with the 2D-Cardiac Performance Analysis module. GLS and SR were derived using Image-Arena v4.6 software (TomTec Imaging System). GLS values were computed if > 14 of 17 segments were adequately tracked. In patients with atrial fibrillation (AF), the index beat method was used to ensure consistency. Preoperative RV function was assessed by means of transthoracic echocardiography using the peak systolic velocity of the tricuspid annulus, measured by pulsed-wave Doppler tissue imaging (DTI) (s’ in cm/s), and the tricuspid annular plane systolic excursion (TAPSE), measured by M-mode (in mm) before cardiac surgery. Pulmonary artery systolic pressures (sPAP) were estimated using Doppler echocardiography when tricuspid regurgitation was detected [14]. Further details on echocardiographic measurements and assessment of left and right ventricular function are presented in the Supplementary Material (*Supplementary material, Supplemental material 1, Sect. 1.1 Echocardiography*).

### Study endpoints

Primary outcomes were as follows: (i) the occurrence of postoperative LCOS defined as the evidence of inadequate organ perfusion which required sustained inotropic medication, vasopressors, or mechanical circulatory support devices for at least 24 h; and (ii) the identification of a GLS and SR value threshold associated with LCOS after mitral valve surgery.

Predefined secondary outcomes included postoperative acute kidney injury according to the KDIGO criteria [15], postoperative hepatic disfunction (defined as bilirubin > 3 mg/dL, duration of mechanical ventilation, length of ICU and hospital stay, readmission in intensive care unit, 30-day and 1-year all-cause mortality). Telephonic 30-day and 1-year follow-up were performed to investigate mortality and re-hospitalization due to heart failure.

### Statistical analyses

#### Sample size calculation

Previous investigations have reported that impaired GLS and SR were associated with worse short- and long-term outcomes [16]. Estimating a 20% incidence of LCOS after on-pump mitral surgery [17] and anticipating a risk of LCOS at 35% in patients with impaired GLS, we calculated that a sample size of 122 patients would be required to detect the difference with 12% absolute precision and a 95% confidence level. To account for an expected 10% dropout rate, final sample size was increased to 134 and rounded up to 140.

#### Primary analysis

All statistical analyses were conducted using R Statistical Software (version 4.1.1, Foundation for Statistical Computing, Vienna, Austria) and MedCalc Statistical Software version 12.7.0 (MedCalc Software by, Ostend, Belgium). Distribution was assessed using the Shapiro–Wilk test. Multiple imputation was employed to handle missing data [18], assuming that the missingness occurred at random. Primary and secondary outcomes showed no missing data and therefore were not imputed. Continuous variables were described using mean or median with corresponding standard deviation (SD) or interquartile range (IQR) and compared with the Mann–Whitney *U* test or *t*-test as appropriate. Categorical variables were presented as counts and percentages and compared with Chi-square analysis or Fisher’s exact test, as needed. The population was dichotomized according to the occurrence of LCOS within 24 h postoperatively. To assess the predictive accuracy of GLS for LCOS after surgery, ROC curves were constructed. The diagnostic efficacy of GLS was evaluated considering sensitivity, specificity, positive predictive value (+ PV), and negative predictive value (NPV). Multivariable logistic regression was used to assess independent predictors of LCOS. Variables with a *p* < 0.05 in univariable analysis were included in a backward stepwise selection process. Model performance was assessed with calibration plots, Hosmer–Lemeshow tests, and c-statistics. Further information on the statistical methods employed for the primary outcomes of the study is available in Supplementary Material (*Supplementary material, Supplemental material 1, Sect. 1.2 Primary Analysis*). Model development utilized the R-package PSFMI, while internal validation and nomogram construction were carried out using the R-package [19, 20]. To facilitate the calculation of risk scores and probabilities for achieving functional recovery, a nomogram was constructed. Two-tailed tests were employed for estimating *p*-values, and statistical significance was defined as a *p*-value < 0.05.

#### Secondary analyses

The stabilized inverse probability of treatment weighting (IPTW) was applied after dichotomizing GLS according to the optimal cutoff value (GLS < − 19.1% or GLS > − 19.1%), to adjust for confounding [21, 22]. Due to missing data, the final IPTW was calculated as an average across all imputed datasets [23]. To assess balance before and after IPTW, the absolute standardized mean difference (ASMD) between groups was calculated, with an ASMD > 0.1 indicating significant imbalance [24]. The GLS effect on primary and secondary outcomes was assessed using the *t*-test and Chi-square test, weighted by the IPTW sample. Average effects of belonging to a GLS < − 19.1% or GLS > − 19.1% were estimated using linear regression models for continuous outcomes and logistic regression models for binary outcomes, with robust estimators. To test the reproducibility of the intraclass correlation coefficients (ICCs), 95% confidence intervals (CIs) were calculated from a randomly selected subset of 40 studies to evaluate both intra-observer and inter-observer variability in GLS measurements. Further details on the IPTW methodology and confounding variables considered are illustrated in Supplementary Material (*Supplementary material, Supplemental material 1, Sect. 1.3 Secondary Analyses*).

## Results

### Primary analysis

A total of 140 patients were screened and 14 were excluded because of poor echocardiographic images (*Supplementary material, Supplemental *Fig. 1) leaving 126 patients to be analyzed.

#### General characteristics of the included population

Patients were 64 (55–73) years old and were predominantly males (56%) (Table 1). The rate of LCOS was 31/126 (25%) and univariate clinical predictors of LCOS were advanced age, history of AF, myocardial infarction, coronary artery disease, and a greater number of diseased coronary arteries. LCOS patients were also more often on preoperative diuretics and oral anticoagulants.

**Table 1:** Demographic and preoperative data

	LCOS	No LCOS	*p*-value
	(*n* = 31)	(*n* = 95)	
Age, years	71.00 [64.5, 77.0]	62.0 [52.5, 71.0]	0.001
BMI, kg/m^2^	24.80 [23.6, 27.2]	24.22 [22.0, 27.3]	0.27
Male, *n* (%)	17 (54.8)	54 (56.8)	1.00
ASA 4 (%)	10 (32.3)	7 (7.4)	0.001
NYHA,* n* (%)			0.03
1	5 (16.1)	24 (25.3)	
2	21 (67.7)	68 (71.6)	
3	5 (16.1)	3 (3.2)	
EuroScore II, (%)	3.41 [2.44, 6.06]	1.26 [0.69, 2.50]	< 0.001
Smoke (%)			0.369
Active	1 (3.2)	10 (10.5)	
Former	6 (19.4)	22 (23.2)	
Myocardial infarction			0.020
< 90 d	2 (6.5)	2 (2.1)	
> 90 d	2 (6.5)	0 (0.0)	
PTCA, *n* (%)	4 (12.9)	6 (6.3)	0.426
Previous, cardiac surgery, *n* (%)			0.250
CABG	0 (0.0)	1 (1.1)	
AVR	0 (0.0)	1 (1.1)	
CAD, *n* (%)	7 (22.6)	7 (7.4)	0.044
CAD numbers, *n* (%)			0.006
1	4 (12.9)	3 (3.2)	
2	1 (3.2)	2 (2.1)	
3	3 (9.7)	0 (0.0)	
4	0 (0.0)	1 (1.1)	
Hypertension, *n* (%)	20 (64.5)	45 (47.4)	0.147
Peripheral vascular disease, *n* (%)	3 (9.7)	6 (6.3)	0.819
Stroke (%)			0.149
1	2 (6.5)	3 (3.2)	
Dyslipidemia, *n* (%)	9 (29.0)	18 (18.9)	0.349
COPD, *n* (%)	3 (9.7)	7 (7.4)	0.976
History of AF, *n* (%)	16 (51.6)	19 (20.0)	0.001
Medications			
Calcium antagonists, *n* (%)	1 (3.2)	10 (10.5)	0.377
RAAS system inhibitors, *n* (%)	15 (48.4)	38 (40.0)	0.286
Withdrawal 24 h before surgery	3 (9.7)	4 (4.2)	
Beta blockers, *n* (%)	19 (61.3)	48 (50.5)	0.518
Withdrawal 24 h before surgery	0 (0.0)	1 (1.1)	
Diuretics	23 (74.2)	38 (40.0)	0.002
Statins, *n* (%)	11 (35.5)	22 (23.2)	0.263
K sparing diuretics, *n* (%)	4 (12.9)	14 (14.7)	1.00
Digoxin, *n* (%)	3 (9.7)	4 (4.2)	0.482
Ranolazine, *n* (%)	1 (3.2)	1 (1.1)	0.99
Aspirin, *n* (%)	8 (25.8)	9 (9.5)	0.061
Withdrawal	0 (0.0)	1 (1.1)	
P_2_Y_12_ inhibitors, *n* (%)	3 (9.7)	1 (1.1)	0.074
Oral anticoagulant, *n* (%)	8 (25.8)	3 (3.2)	< 0.001
New oral anticoagulant, *n* (%)	6 (19.4)	11 (11.6)	0.425
Echocardiographic data			
GLS (%)	− 16.7 [− 19.5, − 15.8]	− 21.7 [− 23.9, − 20.1]	< 0.001
GLS > − 19%, n (%)	23 (74.2)	17 (17.9)	< 0.001
Strain rate, s^−1^	− 1.12 [− 0.88, − 1.30]	− 1.33 [− 1.15, − 1.48]	0.007
Ejection fraction, (%)	56.00 [52.5, 62.0]	63.0 [58.5, 66.0]	< 0.001
TAPSE, mm	19.0 [18.0, 22.0]	24.0 [21.0, 27.0]	< 0.001
DTI s’, cm/sec	11.1 [10.0, 13.0]	14.2 [12.0, 16.9]	< 0.001
sPAP, mmHg	39.0 [30.0, 55.0]	30.0 [25.7, 40.0]	0.007
Mitral regurgitation, n (%)			0.530
1	1 (3.2)	2 (2.1)	
2	2 (6.5)	3 (3.2)	
3	9 (29.0)	19 (20.0)	
4	19 (61.3)	71 (74.7)	
Aortic valve stenosis, *n* (%)			0.718
Moderate	0 (0.0)	1 (1.1)	
Severe	0 (0.0)	1 (1.1)	
Aortic valve regurgitation, *n* (%)			0.005
1	6 (19.4)	15 (15.8)	
2	5 (16.1)	7 (7.4)	
3	5 (16.1)	2 (2.1)	
Tricuspid regurgitation, *n* (%)			0.039
1	8 (25.8)	41 (43.2)	
2	11 (35.5)	26 (27.4)	
3	4 (12.9)	3 (3.2)	
4	4 (12.9)	4 (4.2)	
Diastolic dysfunction			0.676
Impaired relaxation	3 (9.7)	5 (5.3)	
Restrictive pattern	1 (3.2)	4 (4.3)	
Lab tests baseline			
Leukocytes, × 10^3^	6.70 [5.95, 7.90]	6.60 [5.40, 7.55]	0.229
Ht, (%)	40 [38.3, 43.0]	42 [40, 44.1]	0.099
Platelet count, × 10^3^	207 [175.5, 242.5]	206 [180, 249]	0.770
Creatinine clearance, ml/min	54.7 [48.8, 67.6	77.4 [60.3, 100]	< 0.001
Total bilirubin, mg/dl	0.66 [0.53, 1.02]	0.68 [0.47, 0.97]	0.799
Direct bilirubin, mg/dl	0.26 [0.20, 0.37]	0.25 [0.18, 0.36]	0.716
AST, U/l	26.0 [20.0, 31.5]	23.0 [20.0, 28.0]	0.439
ALT, U/l	22.0 [18.0, 31.0]	24.0 [18.0, 32.0]	0.538
INR	1.17 [1.06, 1.29]	1.05 [1.00, 1.09]	< 0.001
aPTT, sec	30.0 [27.7, 34.1]	29.9 [28.8, 31.6]	0.810
MCS preoperative, *n* (%)	2 (6.5)	0 (0.0)	0.095

All continuous data are expressed as median with interquartile range [IQR], unless otherwise indicated

No missing data were reported but for the following variables: TAPSE, DTI, sPAP

*Abbreviations*: *LCOS* low cardiac output syndrome, *BMI* body mass index, *ASA* American Society of Anesthesiologists, *NYHA* New York Heart Association, *EUROSCORE* European System for Cardiac Operative Risk Evaluation, *PTCA* percutaneous transluminal coronary angioplasty, *CABG* coronary artery bypass grafting, *AVR* aortic valve replacement, *MV* mitral valve, *CAD* coronary artery disease, *COPD* chronic obstructive pulmonary disease, *AF* atrial fibrillation, *RAAS* renin–angiotensin–aldosterone system, *k* potassium, *GLS* Global Longitudinal Strain, *TAPSE* tricuspid annular Plane systolic excursion, *DTI s’* Doppler tissue imaging systolic wave, *sPAP* systolic pulmonary artery pressures, *RWMA* regional wall motion abnormality, *Ht* hematocrit, *AST* aspartate aminotransferase, *ALT* alanine aminotransferase, *INR* International normalized ratio, *aPTT* activated partial thromboplastin time, *MCS* mechanical circulatory support

#### Baseline echocardiographic data

In terms of baseline echocardiographic data, patients with postoperative LCOS exhibited less negative GLS (− 16.7% [− 19.5, − 15.8] vs − 21.7% [− 23.9, − 20.1]; *p* < 0.001), SR (− 1.12 [− 0.88, − 1.30] vs − 1.33 [− 1.15, − 1.48]; *p* = 0.007), ejection fraction (EF) (56% [52, 62] vs 63% [58, 66]; *p* < 0.001), TAPSE (19 mm [18, 22] vs 24 mm [21, 27]; *p* < 0.001), and DTI s’ (11 cm/s [10, 13] vs 14.2 cm/s [12.0, 16.9]; *p* < 0.001) compared to those without LCOS. Additionally, the LCOS group showed more frequently sPAP above the normal range (*p* = 0.007). Both severe aortic regurgitation and severe tricuspid regurgitation were more frequent in the LCOS group than in the no-LCOS group (respectively, 16.5 and 2.5%, *p* = 0.005; and 13 vs 4%, *p* = 0.039).

#### Baseline laboratory test results and intraoperative data

Baseline lab-test results were similar between LCOS and no-LCOS groups except for a prolonged international normalized ratio (1.17 [1.06, 1.29] vs 1.05 [1.00, 1.09]; *p* < 0.001), and reduced creatinine clearance (54.7 ml/min [48.8, 67.65] vs 77.4 ml/min [60.3, 100.2]; *p* < 0.001). Intraoperative data are summarized in Table 2. The LCOS group exhibited a higher incidence of combined surgery (*p* = 0.001), a greater need for a second run of CPB (*p* = 0.04), and an extended CPB duration (*p* = 0.027) compared to those who did not develop LCOS. Ninety-seven percent of LCOS patients required epinephrine, and 35% of them required intra-aortic balloon pump (IABP) deployment in the operating theater. The LCOS group received a higher dosage of epinephrine (0.10 μg・kg^−1^・min^−1^ [0.05, 0.10] vs 0.05 μg・kg^−1^・min^−1^ [0.03, 0.05]; *p* < 0.001) and was supported with other cardioactive drugs in a greater percentage of cases (*p* = 0.016). Additionally, the transfusion rate was significantly higher in the LCOS group compared to the no LCOS group (23 vs 6%; *p* = 0.025).

**Table 2:** Intraoperative data

	LCOS	No LCOS	*p*-value
	(*n* = 31)	(*n* = 95)	
Surgery, *n* (%)			0.001
Mitral valve repair	7 (22.6)	59.0 (62.1)	
Mitral valve replacement	5 (16.1)	6 (6.3)	
Combined*	19 (61.3)	30 (31.6)	
Second run, *n* (%)	4 (12.9)	2 (2.1)	0.049
CPB time, min	100 [81.5, 137]	87 [70.5, 115]	0.027
Clamping time, min	75 [61.5, 101]	66 [51, 9]	0.061
Epinephrine, *n* (%)	30 (96.8)	75 (78.9)	0.042
μg・kg^−1^・min^−1^	0.10 [0.05, 0.10]	0.05 [0.03, 0.05]	< 0.001
Norepinephrine, *n* (%)	8 (25.8)	22 (23.2)	0.954
μg・kg^−1^・minute^−1^	0.06 [0.05, 0.10]	0.05 [0.05, 0.08]	0.638
PDIII inhibitors, *n* (%)	1 (3.2)	0 (0.0)	0.554
IABP, *n* (%)	11 (35.5)	0	< 0.001
Other cardioactive drugs, *n* (%)			0.016
Methylene blue	0 (0.0)	3 (3.2)	
iNO	2 (6.5)	0 (0.0)	
Other	1 (3.2)	0 (0.0)	
Patients transfused, *n* (%)	7 (22.6)	6 (6.3)	0.025

All continuous data are expressed as median with interquartile range (IQR), unless otherwise indicated

No missing data were reported

*Abbreviations*: *LCOS* low cardiac output syndrome, *CPB* cardiopulmonary bypass, *IABP* intra-aortic balloon pump, *PDIII* phosphodiesterase III

^*^Mitral surgery with at least one of the following procedures: tricuspid valve (*n* = 24); ascending aorta replacement (*n* = 3); David I (*n* = 1); removal of aortic valve mass (*n* = 1); pulmonary vein isolation (*n* = 9); coronary artery bypass grafting (*n* = 5); Morrow (*n* = 1); aortic valve replacement (*n* = 4); left appendage closure (*n* = 6); interatrial defect closure (*n* = 1)

#### Postoperative course

The LCOS group showed a more complicated postoperative course and a significantly higher mortality rate (3 vs 0%; *p* = 0.017) (Table 3). When compared to the no-LCOS group, they required prolonged mechanical ventilation (21 [16.0, 37.7] vs 12.5 h [10.2, 17.2]; *p* < 0.001), higher dosage of epinephrine (0.10 μg・kg^−1^・min^−1^ [0.09, 0.15] vs 0.05 μg・kg^−1^・min^−1^ [0.04, 0.08]; *p* < 0.001), dopamine (μg・kg^−1^・min^−1^ [0, 3] vs 0 μg・kg^−1^・min^−1^ [0, 2]; *p* = 0.009). IABP, phosphodiesterase-III inhibitors (PHIII), and other cardioactive drugs were required in a higher proportion in the LCOS group. These patients received more transfusions (*p* = 0.024) and exhibited higher creatinine levels upon ICU arrival (*p* < 0.001) and during the first postoperative day (*p* < 0.001). Additionally, troponin T levels were higher upon ICU admission (*p* = 0.002), 4 h later (*p* = 0.008), and on the first postoperative day (*p* < 0.001), alongside higher AST upon ICU arrival (*p* = 0.024) and on the first postoperative day (*p* = 0.004). Moreover, although certain postoperative variables (e.g., lactate concentration and urine output at 12 h) differed significantly between groups, the absolute magnitude of these differences was relatively modest (i.e., lactate 3.65 vs 3.04 mmol/L; urine output 1649 vs 2213 mL). Patients in the LCOS group were more often classified as KDIGO 1 and 3 stage (*p* < 0.001), needed non-invasive ventilation (NIV) after 72 h (*p* = 0.002), and experienced cardiogenic shock (*p* < 0.001). The duration of ICU stay was 3 days (2, 5) in the LCOS group and 1 day (1, 1) in the other group.

**Table 3:** Intensive care unit data and postoperative outcomes

	LCOS	No LCOS	*p*-value
	(*n* = 31)	(*n* = 95)	
Mechanical ventilation, h	21.50 [16, 37.7]	12.50 [10.25, 17.25]	< 0.001
Drainage output, ml			
4 h	120 [100, 180]	100 [80, 150]	0.071
12 h	230 [192.5, 287.5]	220 [170, 280]	0.257
24 h	390 [315, 550]	390 [304.50, 462.50]	0.770
Lactate, nmol/l			
ICU admission	4.07 [3.07, 5.33]	3.32 [2.01, 4.75]	0.108
12 h	3.65 [3.20, 5.40]	3.04 [2.02, 4.49]	0.024
24 h	1.70 [1.42, 2.28]	1.40 [0.94, 1.89]	0.026
Urine output, ml	730 [537.5, 101]	970 [754.5, 1380]	0.005
4 h			
8 h	1230 [977.5, 1505]	1669 [1295, 2075]	< 0.001
12 h	1648.50 [1435, 1940]	2212.50 [1745, 2740]	< 0.001
24 h	3440 [2885, 3898.50]	3932 [3210, 4585]	0.056
ICU medications			
Epinephrine*, *n* (%)	31 (100)	83 (87.4)	0.084
μg・kg^−1^・minute^−1^	0.10 [0.09, 0.15]	0.05 [0.04, 0.08]	< 0.001
Norepinephrine*, *n* (%)	12 (38.7)	38 (40.0)	1.00
μg・kg^−1^・minute^−1^	0.10 [0.06, 0.13]	0.05 [0.05, 0.10]	0.052
Dopamine*, *n* (%)	14 (45.2)	11 (11.6)	< 0.001
μg・kg^−1^・minute^−1^	2 [0, 3]	0 [0, 2]	0.009
PHIII inhibitors, *n* (%)	7 (22.6)	0 (0.0)	< 0.001
IABP, *n* (%)	15 (48.4)	0	< 0.001
Other drugs			< 0.001
Nitroprusside	4 (12.9)	0 (0.0)	
Methylene blue	9 (29)	1 (1.1)	
iNO	1 (3.2)	1 (1.1)	
Transfusion, *n* (%)	14 (45.2)	21 (22.1)	0.024
Lab tests at the ICU admission			
Creatinine, mg/dl	1.06 [0.88, 1.21]	0.82 [0.74, 0.94]	< 0.001
Bilirubin total, mg/dl	1.06 [0.77, 1.28]	0.87 [0.62, 1.13]	0.054
Troponin T, ng/ml	816 [541, 1197]	54[365, 719]	0.002
4 h after the admission	1149 [782, 1514]	900 [616, 1137.25]	0.008
Ht, (%)	36 [34.4, 39.4]	36 [33, 38]	0.394
AST, U/l	59 [43.5, 73.25]	48 [39, 61]	0.026
ALT, U/l	19.50 [16.25, 27.5]	19 [14, 27]	0.444
Adverse events			
Myocardial infarction, *n* (%)	1 (3.2)	0 (0.0)	0.554
Cardiogenic shock, *n* (%)	6 (19.4)	0 (0.0)	< 0.001
Respiratory failure, *n* (%)	3 (9.7)	4 (4.2)	0.482
NIV after 72 h, *n* (%)	15 (48.4)	17 (17.9)	0.002
Reintubation, *n* (%)	0 (0.0)	1 (1.1)	1.00
Tracheostomy, *n* (%)	1 (3.2)	0 (0.0)	0.554
KDIGO			< 0.001
1	6 (19.4)	1 (1.1)	
2	0	0	
3	1 (3.2)	0 (0.0)	
New onset of AF, n (%)			0.714
Single episode	6 (19.4)	23 (24.2)	
Persistent	0 (0.0)	1 (1.1)	
Infections, *n* (%)	3 (9.7)	2 (2.1)	0.149
Septic shock	0 (0.0)	1 (1.1)	
Surgical revision, *n* (%)	1 (3.2)	3 (3.2)	1.00
ICU readmission, *n* (%)	1 (3.2)	2 (2.1)	1.00
ICU stay, days	3 [2, 5]	1 [1, 1]	< 0.001
Hospital- stay, days	7 [5, 10]	6 [5, 7]	0.054
In-hospital mortality	1 (3.2)	0 (0.0)	0.554
30-day mortality	0	0	1
1-year mortality	3 (9.7)	0	.017

All continuous data are expressed as median with interquartile range (IQR), unless otherwise indicated

No missing data were reported but for the following variables: ALT, AST, Troponin T, bilirubin total, creatinine, urine output (4, 8, 12 h), lactate (admission, 12 h), drainage output (4, 12, 24 h), mechanical ventilation

*Abbreviations*: *ICU* intensive care unit, *PHIII* phosphodiesterase III, *IABP* intra-aortic balloon pump, *NO* nitric oxide, *NIV* non-invasive ventilation, *KDIGO* kidney disease: improving global outcomes, *AF* atrial fibrillation

^*^Maximum dosage recorded upon ICU admission

#### Definition of GLS and SR optimal cut-offs

According to the Youden index, the optimal threshold for predicting LCOS after surgery was > − 19.1% (95% CI, > − 20– > − 17) for GLS, yielding a sensitivity of 74% (95% CI, 55–88), specificity of 83% (95% CI, 74–90), positive predictive value (+ PV) of 59% (95% CI, 47–70), and negative predictive value (− PV) of 91% (95% CI, 84–95). Similarly, the threshold of ≤ − 0.98 s^−1^ (95% CI, 0.572–0.743) for SR had a sensitivity of 45% (95% CI, 27–64), specificity of 87% (95% CI, 79–93), + PV of 54% (95% CI, 38–69), and − PV of 83% (95% CI, 78–87).

GLS demonstrated moderate performance in distinguishing individuals who suffered from LCOS compared to those who did not, with an AUC of 0.79 (95% CI, 0.71–0.86; *p* < 0.001). In contrast, SR exhibited a lower discriminative value, with an AUC of 0.66 (95% CI, 0.57–0.74; *p* = 0.01) (*Supplementary material, Supplemental *Fig. 2). For EF, the AUC was 0.71 (95% CI, 0.62–0.79; *p* = 0.001) and the best cut-off ≤ 55%.

**Fig. 1:**
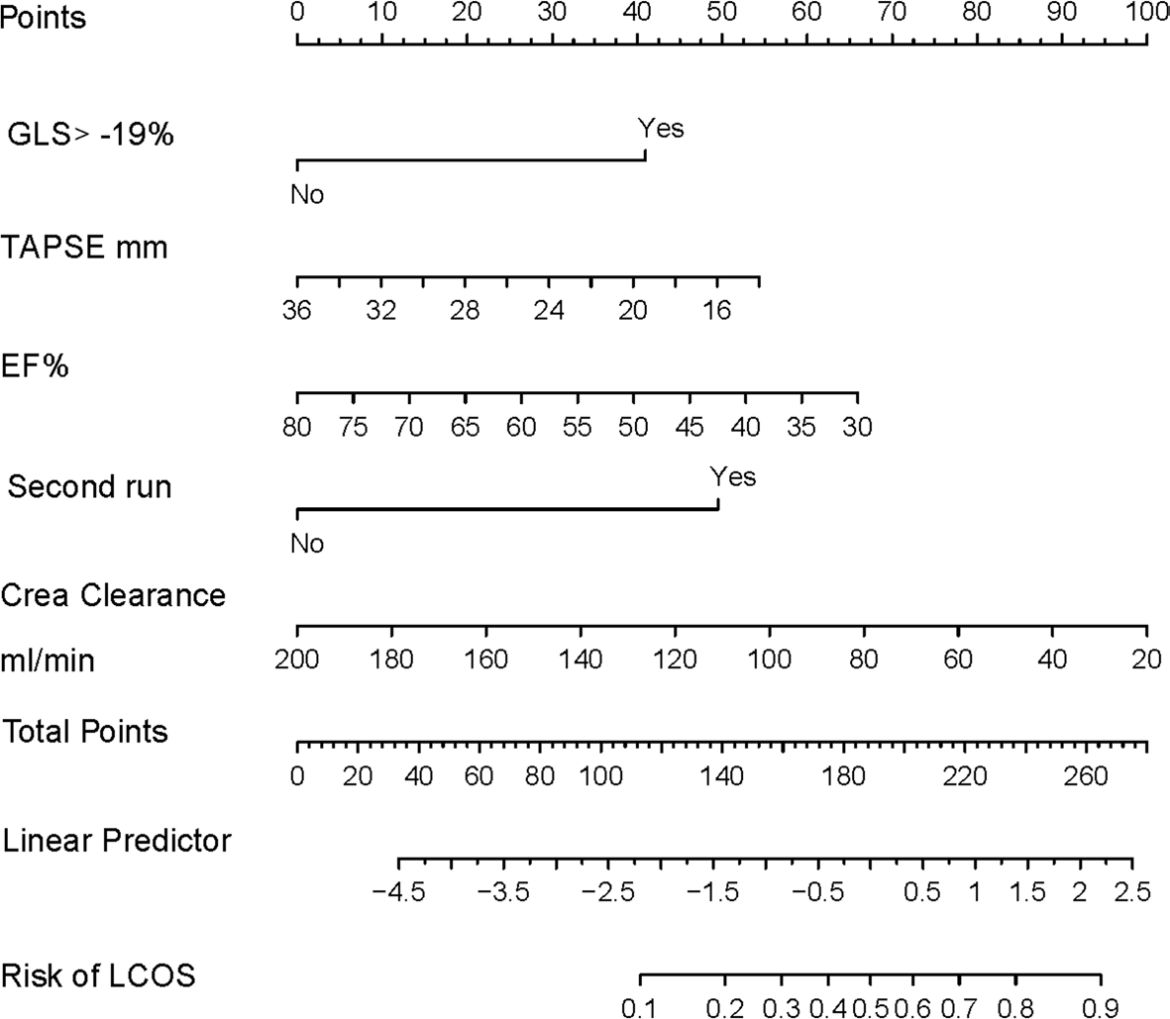
Nomogram for predicting LCOS in mitral surgery patients. The probability of suffering from LCOS can be estimated using the nomogram as follows: (1) assign points to each predictor by drawing a straight line from the predictor to the “Points” line. (2) Sum the assigned points to get a total score (total points line). (3) Determine the likelihood of LCOS by drawing a straight line from the total points line to the “Predicted Value” line

The final model including the significative variables at the univariate analysis identified four predictors for LCOS following mitral surgery: GLS > − 19.1%, TAPSE, EF, and creatinine clearance. Table 4 presents the regression coefficients, standard errors, and odds ratios of these selected predictors. The median Nagelkerke’s *R*^2^ was 0.580 (0.567, 0.582), indicating a substantial explanatory power. The c-statistic, with a median value of 0.898 (0.898, 0.898), reflects excellent discrimination capabilities. The Hosmer–Lemeshow test demonstrated a median *p*-value of 0.257 (0.399, 0.419), suggesting satisfactory calibration. A calibration curve (*Supplementary material, Supplemental Fig. 3*) further illustrated the model’s effective calibration. For internal validation, the median optimism-corrected Nagelkerke’s *R*^2^ stood at 0.517 (0.512, 0.519). The optimism-corrected c-statistic exhibited a median value of 0.77 (0.77; 0.77), indicating acceptable discrimination performance. The optimism-corrected regression coefficients and intercept can be found in the last column of Table 4. A nomogram was created to simplify its application in patients undergoing mitral surgery (Fig. 1). A simulated case example demonstrating the use of the nomogram is reported in the Supplementary Material (*Supplementary material, Supplemental Fig. 4)*.

**Table 4:** Regression coefficients of the apparent and internally validated LCOS prediction model

	LCOS risk development			Internal validation	
	*β*-coefficient	Standard error	*p*-value	Odd ratio	Optimism corrected *β-*coefficient
Intercept	3.172	2.769	0.254	23.86	1.945
GLS < − 19%	2.348	0.616	< 0.001	10.46	1.186
TAPSE, mm	− 0.146	0.072	0.045	0.86	− 0.071
EF, %	− 0.08	0.040	0.033	0.91	− 0.038
Second CPB run	3.168	1.098	0.004	23.76	1.432
Creatinine clearance, ml/min	− 0.030	0.010	0.005	0.97	− 0.016

*Abbreviations*: *LCOS* low cardiac output syndrome, *GLS* global longitudinal strain, *TAPSE* tricuspid annular plane systolic excursion, *EF* ejection fraction, *CPB* cardiopulmonary bypass

### Secondary analyses

**Fig. 2:**
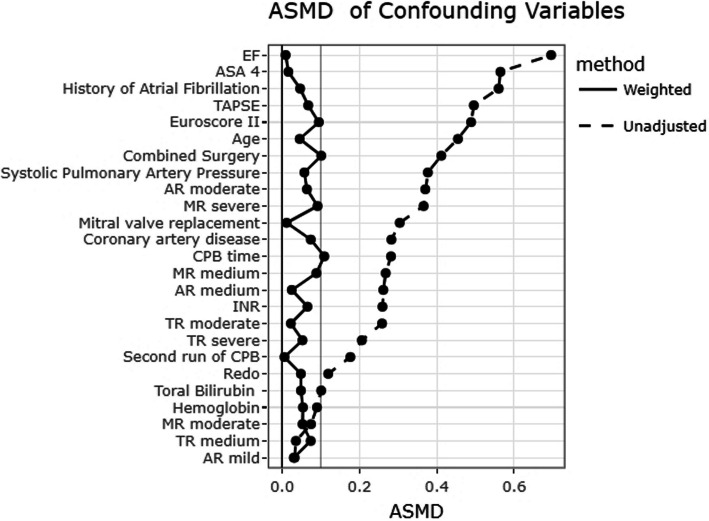
Covariate balance before and after IPTW, evaluated through the absolute standardized mean difference (ASMD). ASA, anesthesiology score assessment; CAD, coronary artery disease; AF, atrial fibrillation; MVR, mitral valve replacement; EF, ejection fraction; TAPSE, tricuspid annular plane systolic excursion; sPAP, systolic pulmonary arterial pressure; TR, tricuspid regurgitation; MR, mitral regurgitation; AR, aortic regurgitation; CPB, cardiopulmonary bypass; INR, International normalized ratio

All 19 preoperative and intraoperative variables reported in *Supplemental *Table 2 were used to construct the IPTW model, which effectively in balanced the selected covariates across the cohort, as shown by improved absolute standardized mean differences (ASMD) (Fig. 2). After IPTW-weighted adjusted analyses, only the association between a preoperative GLS > − 19.1% and postoperative LCOS remained statistically significant (OR 5.48 [95% CI, 1.63–18.5]; *p* = 0.006). Differences in the duration of mechanical ventilation between groups were no longer statistically significant following IPTW adjustment. Additional information on secondary outcomes analyses is available in reported in the Supplementary Material (*Supplementary material, Supplemental material 2, Sect. 2.1 Secondary analyses*).

### Reproducibility analyses

Reproducibility analyses in a subset of 40 randomly selected patients demonstrated excellent reliability of GLS measurements, with high inter- and intra-observer agreement. Additional details are provided in the Supplementary Material (*Supplementary material, Supplemental material 2, Sect. 2.2 Reproducibility analyses*).

## Discussion

### Key findings

TEE-GLS performed in the surgical theater after induction of general anesthesia is the best echocardiographic predictor of postoperative LCOS after cardiac surgery, resulting more useful than the commonly used EF and the emerging TAPSE. Patients with a GLS measurement above − 19.1% faced a threefold higher risk of developing postoperative LCOS, even after adjusting for preoperative clinical and echocardiographic variables. This data was also corroborated after the use of an IPTW to account for the imbalance among the covariates.

Moreover, our findings suggest that—although reduced LVEF and TAPSE were independently associated with postoperative LCOS—their predictive value was inferior to that of GLS. Our results indicate that intraoperative GLS is a reliable indicator of subclinical myocardial dysfunction in patients with mitral disease, and it overcomes intrinsic limitations of LVEF.

Other predictors of LCOS were the need for a second run of CPB which induces myocardial stunning and consequently facilitates the occurrence of postoperative LCOS.

Notably, the cut-off value of GLS > − 19.1% was significantly associated with LCOS, even after adjusting with IPTW for key preoperative factors (e.g., type of surgery, concomitant aortic regurgitation, need for a second CPB run, and CPB duration).

Finally, we corroborated the assumption that renal function plays a determinant role in LCOS, as lower creatinine clearance predisposes patients to a higher risk of experiencing adverse outcomes. This aspect is extensively discussed in the literature [25, 26].

### Relationship with previous literature

Previous studies evaluated the role of TTE GLS and TEE GLS [16, 27, 28], but our study is the first one to evaluate TEE GLS and LCOS after mitral surgery. In their retrospective analysis, Amabili et al. suggested that a GLS obtained from TEE after GA and before CPB of − 17% was significantly associated with LCOS [2]. In contrast to our investigation, which focused exclusively on patients with mitral defects, over 50% of the cardiac population in their study underwent aortic valve replacement or coronary artery bypass graft (CABG) procedures, either isolated or combined. Jha et al. demonstrated that intraoperative GLS measured by TEE in patients with severely depressed LVEF undergoing CABG was predictive of postoperative mortality, need for mechanical circulatory support, or high-dose inotropic support after cardiac surgery [29]. Differently from our study, Jha’s investigation included patients with a LVEF lower than 30% who underwent CABG, which may account for their identification of a remarkably high threshold for TEE-derived LV GLS (− 3%), an unprecedented finding which has never been confirmed thereafter. Sonny et al. found that the TEE GLS and SR correlated with prolonged (i.e., > 7 days) hospital stays but not with the need for postoperative inotropic/vasopressor support or postoperative atrial fibrillation [30]. Notably, they reported a modest AUC (0.67) for GLS and a moderate AUC (0.72) for SR but did not provide specific cutoff values for GLS and SR to identify unfavorable outcomes. Indeed, in experimental models of severe heart failure induced by pressure or volume overload, it has been suggested that changes in GLS/SR may reflect ventricular-arterial coupling rather than intrinsic contractility [31]. In line with this hypothesis, a decrease in GLS/SR, as observed in Sonny’s population of patients with aortic stenosis, would not necessarily indicate reduced LV contractility, while its increase following aortic valve replacement could simply reflect reduced afterload. Conversely, in severe mitral regurgitation, as observed in the present study, in which afterload is already low, even a slight reduction in GLS/SR may signal significantly impaired LV contractility, potentially explaining the marked decrease in GLS/SR after mitral valve repair or replacement. Although this is a compelling hypothesis, further investigation is needed to implement these findings into daily clinical practice.

Notably, Ruppert’s rat model, which employs an acute arteriovenous shunt to induce volume-overload heart failure and transverse aortic constriction to create pressure-overload heart failure, does not accurately replicate the gradual adaptation to volume overload and reduced afterload typically observed in clinical cases of mitral or aortic regurgitation, nor the pressure overload seen in aortic stenosis [31]. Beyond these considerations, this highlights the need for targeted research that includes a homogeneous group of patients, similar to those in the present investigation.

### Significance of study findings

The existing literature consistently suggests a range of preoperative LV GLS values, usually ranging from − 20.9 to − 18%, as predictive markers for postoperative LV dysfunction after cardiac surgery. This range closely aligns with the cutoff of − 19.1% identified in our study to predict LCOS, thereby extending its validity to the perioperative setting, a field that has been relatively neglected in previous investigations [32, 33]. Our research enhances the applicability of LV GLS, even when evaluated via TEE, into the perioperative realm. Our findings underscore that a GLS threshold of − 19.1% may serve as a reliable indicator of early post-operative LCOS, even when evaluated with TEE after GA induction. This critical threshold aids in identifying a specific subset of patients who may benefit from heightened perioperative support and risk management strategies.

The utility of LV GLS as predictor of LCOS over LVEF has a strong pathophysiological background. Unlike LVEF, speckle tracking for myocardial function assessment does not rely on LV volume changes or a geometric model, making it suitable for patients with complex LV deformation. Moreover, studies suggest that longitudinal myocardial function tends to deteriorate earlier than circular function in cardiac disorders, mainly due to its subendocardial localization [28]. Therefore, evaluating longitudinal function rather than radial function—mainly assessed by the LVEF—could aid in the early detection of LV dysfunction—i.e., the main determinant of post-operative LCOS [34]. In the present study focused on mitral surgery, LCOS occurred in 25% of patients, which was in line with data available in published literature [35]. Its occurrence correlated with a more complex perioperative course, with the LCOS group undergoing prolonged mechanical ventilation, necessitating NIV, inotropic agents, or mechanical circulatory support, experiencing cardiogenic shock and renal failure, alongside extended ICU stays and higher 1 year mortality [36]. Thus, pinpointing markers capable of predicting LCOS and initiating early intervention could potentially mitigate this trend. Previously, only Maganti et al. explored the predictors of LCOS following isolated mitral surgery. They reported a 12% incidence of LCOS but a notably higher overall mortality (3.4%). In contrast, our study revealed a higher LCOS incidence (i.e., 25%) but a substantially lower overall in-hospital mortality (i.e., 0.8%). One possible explanation might be that in our study, the prompt administration of treatment for LCOS with inotropes/vasopressors or mechanical circulatory support (MCS) may have prevented prolonged LCOS, which may result in multiorgan failure. Given this, the development of a nomogram—to our knowledge, the first available in the current literature—may support the early prediction of LCOS, thus enabling clinicians to systematically identify patients at higher risk of adverse postoperative functional outcomes. This risk stratification tool could be instrumental in guiding targeted preventive strategies, including the potential use of esmolol as a cardioprotective agent. Esmolol, an ultrashort-acting β1-selective adrenergic blocker, has been shown to modulate the perioperative sympathetic response, reduce myocardial oxygen consumption, and improve left ventricular efficiency, thereby potentially lowering the incidence of LCOS following cardiac surgery [37]. The integration of this nomogram into clinical workflows could help clinicians proactively identify and treat high-risk patients, potentially improving functional recovery and postoperative outcomes.

### Strengths and limitations

The current investigation exhibits both strengths and limitations. Its prospective design and the blinding of both cardiac anesthesiologists and intensivists to the outcomes greatly enhance its validity. Because of the low mortality rate, we were unable to investigate the role of GLS on this outcome. We encountered nearly 10% of cases with insufficient image quality, rendering GLS calculations impossible, the most common issues hindering accurate myocardium tracking and affecting GLS values being acoustic shadowing from mitral valve calcifications, poor lateral wall definition, and low image resolution in the far field. Interestingly, Sonny et al. noted a similar percentage (14%) of cases with inadequate image quality, making intra-operative GLS assessment with TEE unfeasible, aligning closely with our findings in the present study [30]. Notably, in present study, strain data were solely calculated using Philips software. Small variations in strain values may indeed exist among different vendors, emphasizing the need for careful consideration when generalizing findings [38]. Nevertheless, from 2015, there has been a notable enhancement in inter-vendor concordance and reproducibility, surpassing that of traditional echocardiographic parameters such as LVEF and peak Doppler velocities [39]. Although the prevalence of LCOS may seem relatively high, it is consistent with previous studies [2, 17, 35] and may be attributed to the relatively high-risk population under investigation. Although the chosen definition of LCOS may not be the most optimal available, it remains pragmatic, consistent, and reproducible [2, 35]. Additionally, the algorithm for managing difficult weaning from CPB, and more broadly for patients with LCOS, is standardized and has been detailed in a previous publication [12]. Another potential limitation of this analysis is the evaluation of strain parameters after skin incision but before cannulation, as procedures like sternotomy and pericardiotomy could potentially impact GLS/SR acquisition. However, a recent study by Labus et al. specifically examined the effect of these surgical steps on strain measurements, demonstrating that longitudinal strain imaging using TEE remains consistent, reliable, and closely correlates with perioperative GLS measured by TTE [40]. Additionally, Dalla et al. showed that general anesthesia and mechanical ventilation can independently affect the measurement of GLS/SR, likely altering load conditions [41]. Consequently, hemodynamic stability and load conditions are more critical for the accuracy and reliability of GLS/SR comparisons and measurements than the surgical steps themselves [42]. Therefore, another potential limitation of this study is that, while we evaluated LV function using GLS and SR parameters, we did not systematically assess right ventricular function, which is also a well-established determinant of adverse outcomes following cardiac surgery. Thus, this highlights the importance of integrating biventricular assessment and accounting for dynamic changes in preload and afterload when interpreting myocardial deformation parameters in the perioperative setting [43, 44]. Last, we mention that—while certain postoperative clinical and laboratory parameters reached statistical significance (e.g., lactate concentration and urine output at 12 h)—the absolute effect sizes were small. In this regard, we contend that the requirement for inotropes/vasopressors—particularly when considered together alongside signs of hypoperfusion—provides a more accurate characterization of patients with clinically relevant LCOS and thus offers a more comprehensive assessment of the syndrome than reliance on isolated laboratory test differences. Moreover, several factors may account for this observation, spanning the limited follow-up window for laboratory endpoints, the multifactorial nature of postoperative physiology in this patient population, and the possibility that perioperative management strategies (e.g., fluid therapy, vasopressor use) may have attenuated differences between groups. As such, the clinical relevance of these findings may be limited, and larger studies with longer-term outcome assessment are warranted to further clarify their implications.

## Conclusions

In patients undergoing on-pump mitral valve surgery, TEE-GLS obtained pre-CPB is an independent predictor of postoperative LCOS, thus overperforming other echocardiographic measurements. A cutoff value of − 19.1%—even when measured with intraoperative TEE—may offer valuable insights into subclinical preoperative myocardial dysfunction, thus potentially enhancing prognostic accuracy and informing perioperative management strategies.

## Supplementary Information

Supplementary Material 1.

## Data Availability

Further information is available from the corresponding authors upon reasonable request.
