# Supplementary material for: Intraoperative global longitudinal strain and strain rate as predictors of unfavorable outcome following on-pump mitral surgery: a prospective observational study
Source: J Anesth Analg Crit Care. 2025 Oct 22;5:70. doi: 10.1186/s44158-025-00288-1 (PMC12542371; doi:10.1186/s44158-025-00288-1)
Supplement: Supplementary file 1 — Supplementary Material 1. [file 44158_2025_288_MOESM1_ESM.doc]

**Supplementary Material to:**

Intraoperative Global Longitudinal Strain and Strain Rate as Predictors of Unfavorable Outcome in On-Pump Mitral Surgery. A Prospective Observational Study.

**Table of Contents**

| **Affiliations of the Mit-Go collaborators** |  | p. 2 |
| --- | --- | --- |
| **Supplemental Material 1.** | Additional methodological considerations | pp. 3-7 |
| **Supplemental Material 2.** | Additional considerations on the results of the secondary and reproducibility analyses | pp. 8-9 |
| **Supplemental Figure 1.** | Flow-chart for patient enrolment | p. 10 |
| **Supplemental Figure 2.** | Receiver Operating characteristics curves | p. 11 |
| **Supplemental Figure 3.** | Calibration curve | p. 12 |
| **Supplemental Figure 4.** | Step-by-step guide to nomogram interpretation | p. 13 |
| **Supplemental Table 1.** | STROBE checklist | pp. 14-15 |
| **Supplemental Table 2.** | Unadjusted and adjusted preoperative and intraoperative variables for 126 patients based on GLS category | p. 16 |
| **Supplemental Table 3.** | Adjusted and unadjusted postoperative outcomes | p. 17 |

**Affiliations of the Mit-Go collaborators**

**Mit-Go Study Group Non-author Collaborators (name, surname):**

Giuseppe Giardina, RN^1^, Cristina Nakhnoukh, MS^1^, Samuele Bugo, MD^1^, Emanuele Ghirardi, MDs^2^, Beatrice Righetti, MD^1^, Viviana Teresa Agosta, MD^1^, Alice Bottussi, MD^1^

**Collaborators’ affiliations**

*^1^ Department of Cardiothoracic and Vascular Anesthesia, IRCCS San Raffaele Scientific Institute, Milan, Italy.*

*^2^ School of Medicine, Vita-Salute San Raffaele University, Milan, Italy*

**Supplemental Material 1.** Additional methodological considerations.

**1.1 Echocardiography**

The endocardial borders were delineated in the end-systolic frame, forming a region of interest after manual adjustments of the epicardial borders. The peak of the R wave was employed as a reference point to identify end-diastole, and the time to aortic closure was measured using the mid-esophageal long-axis view. Manual adjustments of the region of interest were made as needed to optimize myocardial tracking. The tracking's integrity was visually inspected, and GLS was computed only if at least 14 of the 17 segments were adequately tracked. As per recommendations, changes in GLS were described using absolute values, signifying that a decreased GLS indicates a less negative value, signaling a deterioration in left ventricular function. In patients with AF, the index beat assessment has been employed to address heart rate inter-beat variability. This method entails selecting appropriate cardiac cycles that demonstrate nearly equal preceding and pre-preceding RR intervals, along with similar RR intervals [1,2]. This pragmatic approach is recognized as both reliable and reproducible [3], providing an effective alternative to the time-consuming method of averaging echocardiographic parameters over at least five beats, as recommended by the guidelines on cardiac chamber quantification for patients with atrial fibrillation [4]. Left ventricular function was assessed by calculating the end systolic volume, end diastolic volume and biplane ejection fraction using the Simpson’s rule in the mid esophageal four chamber view and two chamber view. Preoperative right ventricular function was assessed using the peak systolic velocity of the tricuspid annulus, measured by pulsed-wave Doppler tissue imaging (DTI) (s' in cm/sec), and the tricuspid annular plane systolic excursion (TAPSE), measured by M-mode (in mm). Pulmonary artery systolic pressures (sPAP) were estimated using Doppler echocardiography when tricuspid regurgitation was detected [5]. According with the current evidence abnormal sPAP was defined as an estimated sPAP above 35 mmHg [5,6].

**1.2 Statistics**

*1.2.1 Imputation*

For optimal imputation accuracy of missing data, predictions were based on the outcome variable, all potential predictors, and auxiliary variables exhibiting a correlation of at least 0.4 with the variable being imputed [7]. Continuous variables were imputed using predictive mean matching, while binary and categorical variables were imputed using logistic, respectively [7]. The determination of the number of imputed datasets followed Von Hippel's approach, using a coefficient of variation of .05. A total of five imputed datasets were generated through 50 iterations [8].

*1.2.2 Best cut-off*

The optimal threshold for ROC curves was determined using the Youden index (J = sensitivity - specificity - 1). The Area Under the Curve (AUC) and its exact binomial 95% confidence interval (CI) were computed.

*1.2.3 Full Model predictors*

Multivariable logistic regression analysis was employed to assess the association of predictors with the outcome in each imputed dataset. The full model considered all predictors with a p value < .05 at the univariable analysis and underwent elimination through backward stepwise selection. The following variables have been included in the analysis: GLS (dichotomized according to the ROC curve), SR, age, EuroScore II, previous myocardial infarction, number of diseased coronary artery vessels, history of AF, diuretic use, NYHA class, TAPSE, DTI, EF, sPAP, INR, aortic regurgitation, type of surgery, second run of CPB, CPB duration, and creatinine clearance. Determination of the predictors to be retained in the final model involved the removal of predictors from the pooled model, utilizing the Pooled Sampling Variance (D1) method [9]. The process was applied independently to each imputed dataset. A p-removal threshold of 0.157 was employed to ensure the inclusion and avoid the exclusion of significant predictors [10]. The final model was aggregated using Rubin’s rules [11,12]. Calibration and apparent performance were assessed through the application of Hosmer-Lemeshow tests in each of the five imputed datasets. A median [Q1; Q3] p-value greater than .05 was deemed indicative of satisfactory calibration [12]. Additionally, calibration plots were generated for each dataset. The apparent performance was analyzed by calculating the median [Q1; Q3] Nagelkerke’s R^2^ and c-statistic across the imputed datasets [12]. A c-statistic value of 0.5 indicates no discrimination beyond random chance. The criteria for interpreting the c-statistic are as follows: 0.7 ≤ c-statistic < 0.8 is considered 'acceptable discrimination,' 0.8 ≤ c-statistic < 0.9 is categorized as 'excellent discrimination,' and c-statistic ≥ 0.9 is regarded as 'outstanding discriminatio' [13].

*1.2.4 Internal validation and adjusted performance*

Internal validation and adjusted performance metrics were applied to the developed model through bootstrap resampling. In each dataset, 200 bootstrap samples were drawn, and within each sample, the model underwent re-estimation using backward stepwise selection [14]. Then, the optimism-corrected performance was computed as the difference between apparent performance and optimism: Optimism-corrected performance = apparent performance – optimism [15]. The optimism-corrected coefficients and intercepts were calculated and subsequently aggregated using Rubin’s Rules [11,12]. The median [Q1; Q3] optimism-corrected Nagelkerke’s R^2^ and c-statistic were then determined [12]. To facilitate the calculation of risk scores and probabilities for achieving functional recovery, a nomogram was constructed.

**1.3 Secondary analyses**

*1.3.1 Inverse probability of treatment weighting (IPTW)*

We controlled for available baseline and surgical potential confounding variables, including age, ASA 4, EuroScore II, coronary artery disease, history of AF, Redo, surgery, EF, TAPSE, sPAP, degree of tricuspid, mitral and aortic regurgitation, CPB time, second run of CPB, INR, total Bilirubin, hemoglobin. The stabilized inverse probability of treatment weighting (IPTW) method comprises two key steps: i) estimating a propensity score for each patient, which indicates the likelihood of having a low GLS. This is derived from a multivariable logistic regression model in which GLS > -19,1% serves as the dependent variable, while baseline potential confounding variables function as explanatory variables; and ii) assigning a propensity score weight to each patient to evaluate the hypothesized association between GLS categories and study endpoints. This IPTW approach allocates greater weight to patients with normal GLS who closely resemble those with low GLS, while assigning less weight to those who are less similar. This strategy aims to optimize the balance between patients with low GLS and those with normal GLS regarding potential confounders following the weighting process.

**Supplemental References**

1. Donal E, Lip GYH, Galderisi M, Goette A, Shah D, Marwan M, et al. EACVI/EHRA Expert Consensus Document on the role of multi-modality imaging for the evaluation of patients with atrial fibrillation. European Heart Journal - Cardiovascular Imaging. 2016;17:355–83.

2. Kotecha D, Mohamed M, Shantsila E, Popescu BA, Steeds RP. Is echocardiography valid and reproducible in patients with atrial fibrillation? A systematic review. EP Europace. 2017;19:1427–38.

3. Hagendorff A, Stöbe S, Helfen A, Knebel F, Altiok E, Beckmann S, et al. Echocardiographic assessment of atrial, ventricular, and valvular function in patients with atrial fibrillation-an expert proposal by the german working group of cardiovascular ultrasound. Clin Res Cardiol. 2024;

4. Lang RM, Badano LP, Mor-Avi V, Afilalo J, Armstrong A, Ernande L, et al. Recommendations for cardiac chamber quantification by echocardiography in adults: an update from the American Society of Echocardiography and the European Association of Cardiovascular Imaging. J Am Soc Echocardiogr. 2015;28:1-39.e14.

5. Greiner S, Jud A, Aurich M, Hess A, Hilbel T, Hardt S, et al. Reliability of Noninvasive Assessment of Systolic Pulmonary Artery Pressure by Doppler Echocardiography Compared to Right Heart Catheterization: Analysis in a Large Patient Population. Journal of the American Heart Association. 2014;3:e001103.

6. Essayagh B, Benfari G, Antoine C, Grigioni F, Le Tourneau T, Roussel J-C, et al. Reappraisal of the Concept and Implications of Pulmonary Hypertension in Degenerative Mitral Regurgitation. JACC Cardiovasc Imaging. 2024;S1936-878X(24)00195-5.

7. Buuren S van. Flexible Imputation of Missing Data, Second Edition. 2nd ed. New York: Chapman and Hall/CRC; 2018.

8. Si Y, Heeringa S, Johnson D, Little RJA, Liu W, Pfeffer F, et al. Multiple Imputation with Massive Data: An Application to the Panel Study of Income Dynamics. J Surv Stat Methodol. 2023;11:260–83.

9. Li KH, Raghunathan TE, Rubin DB. Large-Sample Significance Levels from Multiply Imputed Data Using Moment-Based Statistics and an F Reference Distribution. Journal of the American Statistical Association. 1991;86:1065–73.

10. Collins GS, Reitsma JB, Altman DG, Moons KGM. Transparent reporting of a multivariable prediction model for individual prognosis or diagnosis (TRIPOD): the TRIPOD statement. BMJ. 2015;350:g7594.

11. Rubin DB, Schenker N. Multiple imputation in health-care databases: an overview and some applications. Stat Med. 1991;10:585–98.

12. Marshall A, Altman DG, Holder RL, Royston P. Combining estimates of interest in prognostic modelling studies after multiple imputation: current practice and guidelines. BMC Med Res Methodol. 2009;9:57.

13. Hosmer Jr. DW, Lemeshow, S, Sturdivant, RX. Applied Logistic Regression, 3rd Edition | WileyVol. (2013) 398, John Wiley & Sons. [Internet]. Wiley.com. 2013 [cited 2024 Jan 23]. Available from: https://www.wiley.com/en-gb/Applied+Logistic+Regression%2C+3rd+Edition-p-9780470582473

14. Harrell , FE. Regression Modeling Strategies: With Applications to Linear Models, Logistic and Ordinal Regression, and Survival Analysis [Internet]. Cham: Springer International Publishing; 2015 [cited 2024 Jan 23]. Available from: https://link.springer.com/10.1007/978-3-319-19425-7

15. Steyerberg EW. Clinical Prediction Models: A Practical Approach to Development, Validation, and Updating. Springer International Publishing; 2019.

**Supplemental Material 2.** Additional considerations on the results of the secondary and reproducibility analyses.

**2.1 Secondary analyses**

All 19 preoperative and intraoperative variables reported in *Supplemental Table 2* were used to construct the IPTW model. The IPTW was highly effective in balancing the selected covariates across the cohort, as demonstrated by the improvement in absolute standardized mean differences (ASMD) (*Figure 2*). Before applying IPTW, 14 of the 19 variables had an ASMD greater than 0.1, indicating imbalance. After propensity score adjustment, these differences were eliminated (*Supplementary Material, Supplemental Table 2*).

The clinical outcomes prior to and following IPTW are detailed in *Supplemental Table 3* which provides a summary of both unweighted and IPTW-weighted adjusted occurrences of the primary and secondary clinical outcomes (*Supplementary Material, Supplemental Table 3*). The crude estimates for the incidence of LCOS demonstrated a significantly higher proportion of patients with a preoperative GLS of > -19,1% compared to those with a GLS of -19,1% or lesser (OR: 13.19 [95% CI: 5.03–34.6]; *P* < .0001). Additionally, the crude estimates indicated that the duration of mechanical ventilation was significantly longer in patients with a GLS of greater than -19,1% compared to those with a GLS of -19,1% or lesser [MD: 6.54 hours (95% CI, 12.79-0.29; *P*=.04). After conducting IPTW-weighted adjusted analyses, patients with a GLS of greater than -19,1% were found to have only higher odds of developing postoperative LCOS compared to those without this GLS value (OR: 5.48 [95% CI, 1.63–18.5]; p=.006). The duration of mechanical ventilation was no longer significant different between the 2 groups after IPTW-weighted adjustment.

**2.2 Reproducibility analyses**

Reproducibility analyses were conducted on a randomly selected subset of 40 patients. High Intraclass Correlation Coefficients (ICCs) were observed for GLS. For inter-observer reliability, the ICC for single measures was 0.92 (95% CI, 0.85–0.95), indicating strong agreement between different raters. For average measures, the ICC increased to 0.96 (95% CI, 0.92–0.98), reflecting even higher consistency when averaging the measurements. Similarly, intra-observer reliability showed excellent consistency, with an ICC of 0.96 (95% CI. 0.91–0.98), highlighting strong reproducibility within repeated measurements by the same observer.

**Supplemental Figure 1.** Flow-chart for patient enrolment.

Patients assessed for eligibility

n=214

Patients not included in the study (n=74)

- Declined to participate: 33
- Redo with mechanical mitral valve: 10
- Age < 18 years old: 2
- Urgent surgery: 12
- TEE images non available: 27

Patients enrolled

n=140

Inadequate TEE images resolution (n=14)

 ECG issues: 3

 Lack of at least one view among ME long axis, two chamber, or four chamber: 6

 More than 3 segments optimally trackable: 5

Patients analyzed

n=126

LCOS

n=31

NO LCOS

n=95

TEE: Transesophageal Echocardiography; ECG: Electrocardiogram; ME:Mid-esophageal; LCOS: Low Cardiac Output Syndrome.

**Supplemental Figure 2.** Receiver Operating characteristics curves (ROC)


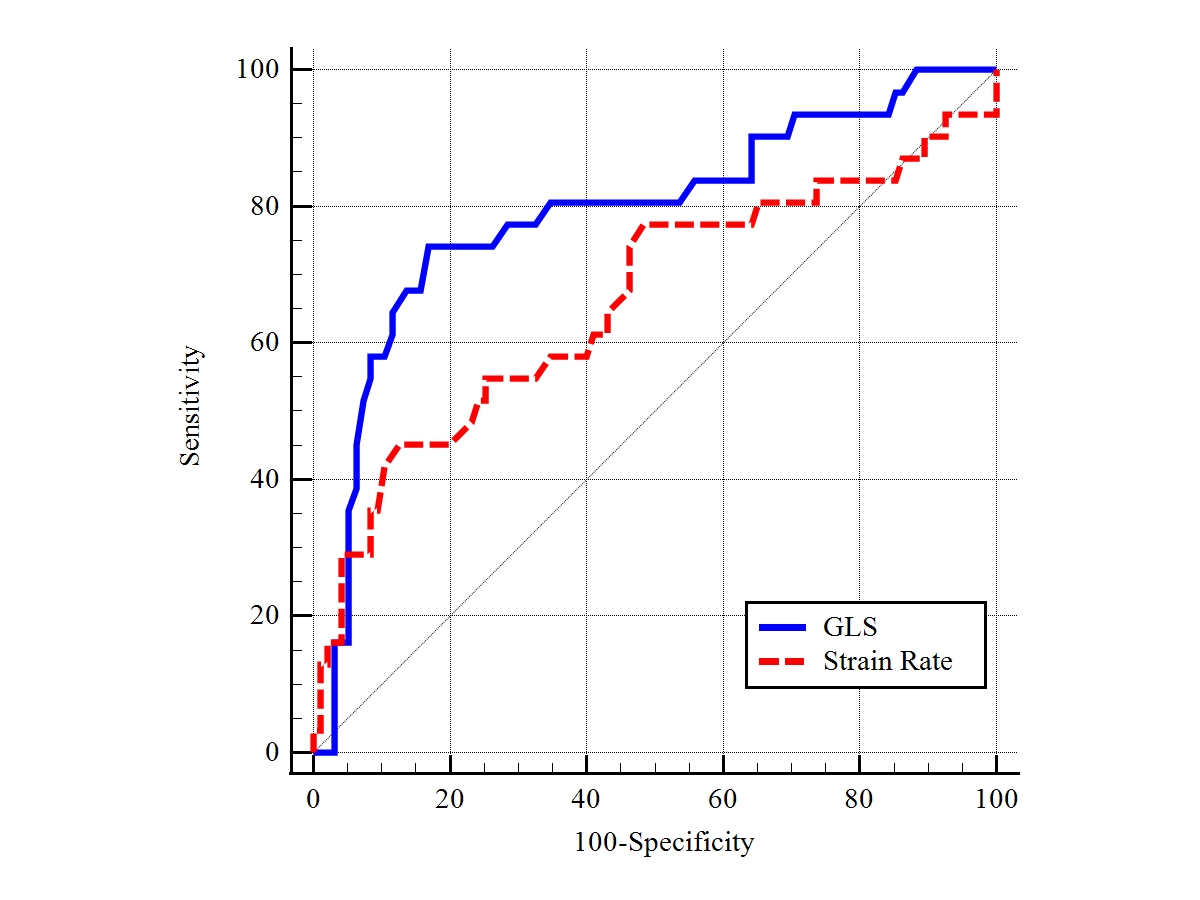


Receiver operating Characteristic curves analysis. GLS: Global Longitudinal Strain.

**Supplemental Figure 3.** Calibration curve

**
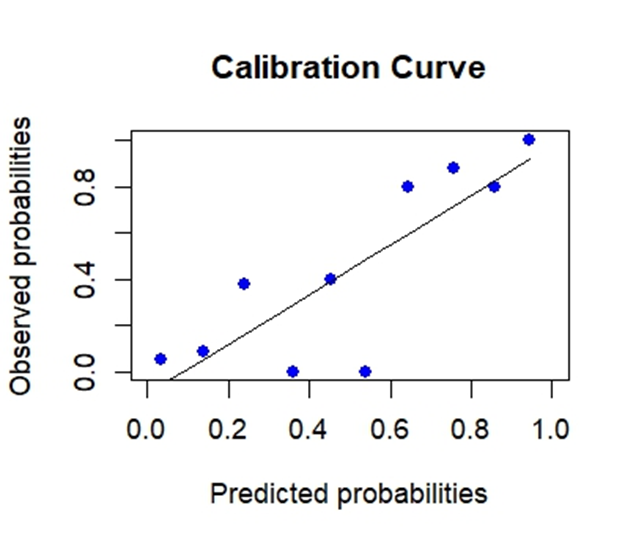
**

The figure shows the calibration curve for the final model. The solid black line represents perfect calibration, while the blue dots represent the actual calibration of the model.

**Supplemental Figure 4.** Nomogram interpretation step-by-step guide


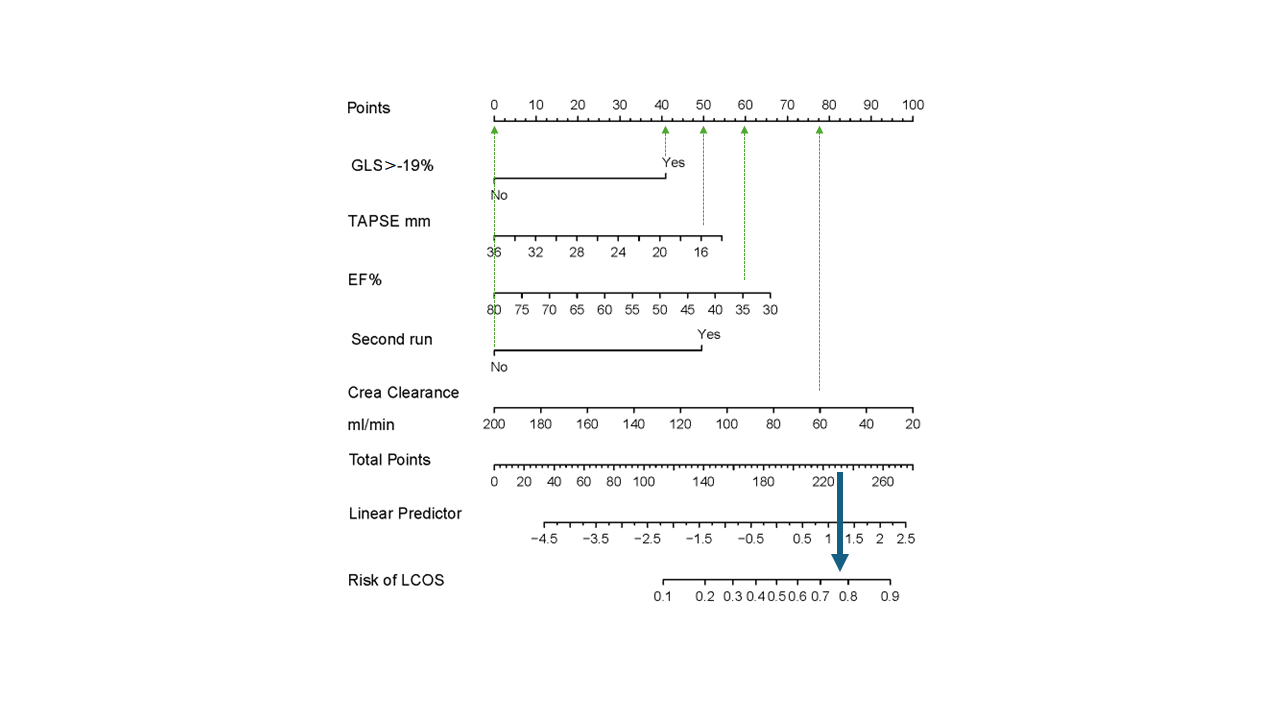


To estimate the probability of LCOS after mitral valve surgery using the nomogram, follow these steps:

1. Assign Points Based on Predictor Values:
   - For GLS > -19.1%, draw a straight line up to the "Points" line. This value gives 40 points (indicated by the green dotted line).
   - For TAPSE = 16 mm, draw a straight line to the "Points" line. This gives 50 points (red dotted line).
   - For EF = 35%, draw a line to the "Points" line, assigning 60 points (purple dotted line).
   - For Creatinine Clearance = 60 mL/min, this gives 77.5 points (yellow dotted line).
2. Sum the Points:
   - Add the individual points: 40 (GLS) + 50 (TAPSE) + 60 (EF) + 77.5 (Creatinine Clearance) = 225 total points.
3. Determine the Predicted Probability:
   - Draw a straight line from the "Total Points" value (225 points) down to the "Predicted Value" line. This gives the probability of LCOS.
   - In this clinical example, the predicted probability of suffering from LCOS after surgery is 0.77, or a 77% chance.

This method gives a visual and quantitative prediction for LCOS based on the values of key predictors.

**Supplemental Table 1.** STROBE Statement—Checklist of items that should be included in reports of *cohort studies*

|  | Item No | Recommendation | Page  No |  |  |
| --- | --- | --- | --- | --- | --- |
| **Title and abstract** | 1 | (*a*) Indicate the study’s design with a commonly used term in the title or the abstract | 1 |  |  |
|  |  | (*b*) Provide in the abstract an informative and balanced summary of what was done and what was found | 1 |  |  |
| Introduction | | |  |  |  |
| Background/rationale | 2 | Explain the scientific background and rationale for the investigation being reported | 1 |  |  |
| Objectives | 3 | State specific objectives, including any prespecified hypotheses | 2 |  |  |
| Methods | | |  |  |  |
| Study design | 4 | Present key elements of study design early in the paper | 3 |  |  |
| Setting | 5 | Describe the setting, locations, and relevant dates, including periods of recruitment, exposure, follow-up, and data collection | 3-5 |  |  |
| Participants | 6 | (*a*) Give the eligibility criteria, and the sources and methods of selection of participants. Describe methods of follow-up | 5 |  |  |
|  |  | (*b*) For matched studies, give matching criteria and number of exposed and unexposed |  |  |  |
| Variables | 7 | Clearly define all outcomes, exposures, predictors, potential confounders, and effect modifiers. Give diagnostic criteria, if applicable | 5 |  |  |
| Data sources/ measurement | 8* | For each variable of interest, give sources of data and details of methods of assessment (measurement). Describe comparability of assessment methods if there is more than one group | 5-7 |  |  |
| Bias | 9 | Describe any efforts to address potential sources of bias | 7 |  |  |
| Study size | 10 | Explain how the study size was arrived at | 5 |  |  |
| Quantitative variables | 11 | Explain how quantitative variables were handled in the analyses. If applicable, describe which groupings were chosen and why | 6 |  |  |
| Statistical methods | 12 | (*a*) Describe all statistical methods, including those used to control for confounding | 6-7 |  |  |
|  |  | (*b*) Describe any methods used to examine subgroups and interactions | 6-7 |  |  |
|  |  | (*c*) Explain how missing data were addressed | 7 |  |  |
|  |  | (*d*) If applicable, explain how loss to follow-up was addressed |  |  |  |
|  |  | (*e*) Describe any sensitivity analyses | NA |  |  |
| Results | | |  |  |  |
| Participants | 13* | (a) Report numbers of individuals at each stage of study—eg numbers potentially eligible, examined for eligibility, confirmed eligible, included in the study, completing follow-up, and analysed | 8 and  Supplemental material Figure 1 |  |  |
|  |  | (b) Give reasons for non-participation at each stage | Supplemental material Figure 1 |  |  |
|  |  | (c) Consider use of a flow diagram | Supplemental material Figure 1 |  |  |
| Descriptive data | 14* | (a) Give characteristics of study participants (eg demographic, clinical, social) and information on exposures and potential confounders | 8 |  |  |
|  |  | (b) Indicate number of participants with missing data for each variable of interest | 8-10 |  |  |
|  |  | (c) Summarise follow-up time (eg, average and total amount) | 14 |  |  |
| Outcome data | 15* | Report numbers of outcome events or summary measures over time | 8-14 |  |  |
| Main results | 16 | (*a*) Give unadjusted estimates and, if applicable, confounder-adjusted estimates and their precision (eg, 95% confidence interval). Make clear which confounders were adjusted for and why they were included | 8-14 |  |  |
|  |  | (*b*) Report category boundaries when continuous variables were categorized | 8-14 |  |  |
|  |  | (*c*) If relevant, consider translating estimates of relative risk into absolute risk for a meaningful time period | NA |  |  |
| Other analyses | 17 | Report other analyses done—eg analyses of subgroups and interactions, and sensitivity analyses |  |  |  |
| Discussion | | |  |  |  |
| Key results | 18 | Summarise key results with reference to study objectives | 15-16 |  |  |
| Limitations | 19 | Discuss limitations of the study, taking into account sources of potential bias or imprecision. Discuss both direction and magnitude of any potential bias | 19-21 |  |  |
| Interpretation | 20 | Give a cautious overall interpretation of results considering objectives, limitations, multiplicity of analyses, results from similar studies, and other relevant evidence | 16-19 |  |  |
| Generalisability | 21 | Discuss the generalisability (external validity) of the study results | 21 |  |  |
| Other information | | |  |  |  |
| Funding | 22 | Give the source of funding and the role of the funders for the present study and, if applicable, for the original study on which the present article is based | Title page |  |  |

*Give information separately for exposed and unexposed groups.

**Note:** An Explanation and Elaboration article discusses each checklist item and gives methodological background and published examples of transparent reporting. The STROBE checklist is best used in conjunction with this article (freely available on the Web sites of PLoS Medicine at http://www.plosmedicine.org/, Annals of Internal Medicine at http://www.annals.org/, and Epidemiology at http://www.epidem.com/). Information on the STROBE Initiative is available at [http://www.strobe-statement.org](http://www.strobe-statement.org/).

| **Supplementary Table 2.** Unadjusted and Adjusted preoperative and intraoperative variables for 126 patients based on GLS category. | | | | | | | | |
| --- | --- | --- | --- | --- | --- | --- | --- | --- |
|  |  | **Before ITPW** | | |  | **After ITPW** | | |
|  |  | **GLS < 19.1%** | **GLS ≥-19.1%** | **p-value** |  | **GLS < 19.1% %** | **GLS ≥-19.1** | **p-value** |
|  |  | (n=40) | (n=86) |  |  | (n=43.5) | (n=82.5) |  |
| Age, years |  | 66.7 ± 11.7 | 61.1± 12.7 | .021 |  | 61.9 ±12 | 62.4 ± 12.3 | .084 |
| ASA 4, n (%) |  | 11 (27.5) | 6 ( 7.0) | .004 |  | 5.2 (11.9) | 10.3 (12.5) | .094 |
| EuroScore II mortality, (%) |  | 3.68 ± 3.17 | 2.26 ±2.6 | .009 |  | 2.33 ± 2.69 | 2.6 ± 3.04 | .065 |
| Coronary artery disease, n (%) |  | 7 (17.5) | 7 ( 8.1) | .021 |  | 3.9 ( 8.9) | 9.2 (11.1) | .070 |
| Atrial Fibrillation, n (%) |  | 18 (45.0) | 17 (19.8) | .006 |  | 9.1 (20.9) | 18.9 (22.9) | .081 |
| Redo, n (%) |  | 3 ( 7.5) | 4 ( 4.7) | .082 |  | 1.7 ( 3.8) | 4.0 ( 4.8) | .077 |
| Mitral valve replacement, n (%) |  | 6 (15.0) | 5 ( 5.8) | .017 |  | 3.0 ( 7.0) | 5.5 ( 6.7) | .094 |
| Combined surgery, n (%) |  | 21 (52.5) | 28 (32.6) | .052 |  | 14.7 (33.8) | 31.9 (38.7) | .066 |
| Ejection fraction, % |  | 56.9 ± 9.38 | 62.5 ± 6.41 | <.001 |  | 61.4 ± 7.80 | 61.4 ± 7.28 | .097 |
| TAPSE, mm |  | 21.7 ± 4.37 | 23.9 ± 4.42 | .013 |  | 23.8 ± 4.28 | 23.5 ± 4.49 | .077 |
| sPAP, mmHG |  | 39.1 ± 11.8 | 34.3 ±13.2 | .094 |  | 35.1 ± 10.2 | 34.4 ± 13.1 | .078 |
| TR: moderate, n (%) |  | 15 (37.5) | 22 (25.6) | .025 |  | 13.1 (30.0) | 23.9 (29.0) | .922 |
| TR: medium, n (%) |  | 2 ( 5.0) | 5 ( 5.8) | 1.00 |  | 1.7 ( 4.0) | 4.6 ( 5.5) | .070 |
| TR: severe, n (%) |  | 4 (10.0) | 4 ( 4.7) | .045 |  | 2.1 ( 4.9) | 5.0 ( 6.1) | .077 |
| MR: moderate, n (%) |  | 2 ( 5.0) | 3 ( 3.5) | 1.00 |  | 1.2 ( 2.8) | 3.1 ( 3.7) | .075 |
| MR: medium, n (%) |  | 12 (30.0) | 16 (18.6) | .023 |  | 8.0 (18.4) | 18.1 (21.9) | .067 |
| MR: severe n (%) |  | 24 (60.0) | 66 (76.7) | .085 |  | 33.4 (76.9) | 60.2 (72.9) | .066 |
| AR: mild, n (%) |  | 7 (17.5) | 14 (16.3) | 1.00 |  | 6.9 (16.0) | 14.1 (17.1) | .089 |
| AR: moderate, n (%) |  | 7 (17.5) | 5 ( 5.8) | .079 |  | 3.4 ( 7.9) | 5.2 ( 6.3) | .071 |
| AR: medium, n (%) |  | 4 (10.0) | 3 ( 3.5) | .029 |  | 2.0 ( 4.5) | 4.2 ( 5.1) | .089 |
| CPB time, minute |  | 104.6 ± 38.9) | 94.5 ± 32.3 | .013 |  | 93.0 ± 35.6 | 96.8 ± 32.9 | .066 |
| Second run of CPB, n (%) |  | 3 ( 7.5) | 3 ( 3.5) | .059 |  | 1.7 ( 3.9) | 3.3 ( 4.0) | .097 |
| INR, |  | 1.18 (0.43) | 1.09 (0.26) | .014 |  | 1.13 (.028) | 1.11 (.028) | .070 |
| Total Bilirubin, mg/dl |  | 0.79 ± 0.53 | 0.75 ± 0.39 | .058 |  | .079 ± .063 | .076 ± .040 | .086 |
| Hemoglobin, g/dl |  | 13.8 ± 1.21 | 14 ± 3.09 | .068 |  | 14.1 ± 1.12 | 14 ± 2.85 | .075 |

All continuous data are expressed as mean ± standard deviation, unless otherwise indicated . GLS: Global Longitudinal Strain; ITPW: Inverse Probability of Treatment Weighting; ASA: Anesthesiology Score Assessment; CAD: Coronary artery disease; AF: Atrial Fibrillation; MVR: Mitral valve replacement; EF: Ejection fraction; TAPSE: Tricuspid annular plane systolic excursion; sPAP: Systolic pulmonary arterial pressure; TR: Tricuspid regurgitation; MR: Mitral regurgitation; AR: Aortic regurgitation; CPB: Cardiopulmonary bypass; INR: International normalized ratio.

| **Supplemental Table 3.** Adjusted and unadjusted postoperative outcomes. | | | | | | | |
| --- | --- | --- | --- | --- | --- | --- | --- |
|  | **Before ITPW** | | |  | **After ITPW** | | |
|  | **GLS < 19.1%** | **GLS ≥-19.1%** | **p-value** |  | **GLS < 19.1% %** | **GLS ≥-19.1** | **p-value** |
|  | (n=40) | (n=86) |  |  | (n=43.5) | (n=82.5) |  |
| LCOS, n (%) | 23 ( 57.5) | 8 ( 9.3) | <.001 |  | 18.9 ( 43.5) | 10.2 ( 12.3) | .005 |
| 1-years Mortality, n (%) | 2 ( 5.3) | 1 ( 1.2) | .046 |  | 1.0 ( 2.4) | 0.9 ( 1.1) | .050 |
| 30 days- Mortality, n (%) | 0 | 0 | NA |  | 0 | 0 | NA |
| ICU readmission, n (%) | 0 ( 0.0) | 3 ( 3.5) | .057 |  | 0.0 ( 0.0) | 2.8 ( 3.4) | .021 |
| Mechanical ventilation time, h | 23.9 ±20.7 | 17.3 ±14.0 | .040 |  | 18 ±14.8 | 18.5 ±15.4 | .085 |
| ICU stay, days | 4.03 ± 8.03 | 2.16 ±6.41 | .016 |  | 2.33 ±4.91 | 2.19 ±6.02 | .087 |
| Hospital stay, days | 8.77 ±5.29 | 6.90 ±4.97 | .058 |  | 8.15 ±6.17 | 7.00 ±4.97 | .043 |
| KDIGO 1, n (%) | 4 ( 10.0) | 3 ( 3.5) | .029 |  | 2.1 ( 4.8) | 2.8 ( 3.4) | .065 |
| KDIGO 3, n (%) | 1 ( 2.5) | 0 ( 0.0) | .069 |  | 0.6 ( 1.4) | 0.0 ( 0.0) | .018 |
| Hepatic injury, n (%) | 1 ( 2.6) | 3 ( 3.5) | 1.00 |  | 0.7 ( 1.7) | 3.3 ( 4.0) | .043 |

All continuous data are expressed as mean ± standard deviation, unless otherwise indicated LCOS: Low Cardiac Output Syndrome; ITPW: Inverse Probability of Treatment Weighting; GLS: Global Longitudinal Strain; ICU: Intensive Care Unit; KDIGO: Kidney Disease: Improving Global Outcomes.
